# Oral Health-related Quality of Life Among 2SLGBTQIAPN+: A Systematic Review

**DOI:** 10.1016/j.identj.2026.109624

**Published:** 2026-05-15

**Authors:** Win Myat Phyo, Aobo Ma, Palinee Detsomboonrat, Juan Ramón Vanegas Sáenz, Duangporn Duangthip, Guang Hong

**Affiliations:** a International Graduate Program in Dental Public Health, Department of Community Dentistry, Faculty of Dentistry, Chulalongkorn University, Bangkok, Thailand; b Division for International Collaborative and Innovative Dentistry, Graduate School of Dentistry, Tohoku University, Sendai, Japan; c Department of Prosthodontics, Faculty of Dental Medicine, Universitas Airlangga, Surabaya, Indonesia; d Department of Community Dentistry, Faculty of Dentistry, Chulalongkorn University, Bangkok, Thailand; e Division of Dental Public Health, College of Dentistry, The Ohio State University, Columbus, Ohio, USA; f Center of Excellence in Precision Medicine and Digital Health, Department of Physiology, Faculty of Dentistry, Chulalongkorn University, Bangkok, Thailand

**Keywords:** Oral health-related quality of life, 2SLGBTQIAPN+, Sexual and gender minorities, Systematic review

## Abstract

Emerging evidence indicates that 2-spirit, lesbian, gay, bisexual, transgender, queer/questioning, intersex, asexual, pansexual, nonbinary and other sexual and gender minorities (2SLGBTQIAPN+) individuals experience disproportionately higher oral disease burdens, which may negatively affect their oral health-related quality of life (OHRQoL). These inequities are associated with financial barriers, lower income, limited affordability of dental care and discrimination, such as misgendering in health care settings. Reviewing OHRQoL in this population is essential to understand the broader impacts of oral health disparities and to guide inclusive, culturally sensitive oral health policies and interventions. A systematic search was conducted on 1 March 2026 across EBSCOhost, PubMed, Scopus and Web of Science, without restrictions on publication year. After removing duplicates, 3 researchers independently performed the selection process, with disagreements resolved through discussion. Google Scholar, reference list screening and citation tracking of included studies were also used to identify additional relevant literature. The inclusion criteria encompassed observational studies that examined OHRQoL among 2SLGBTQIAPN+ individuals using at least 1 validated OHRQoL tool. Six studies met the inclusion criteria. Three were conducted in Brazil and 1 each in India, Malaysia and Australia, with publication dates between 2018 and 2025. Half of the included studies (50%, 3/6) used the Oral Health Impact Profile-14 to measure OHRQoL, with psychological discomfort frequently reported as the most negatively affected domain. Poorer OHRQoL was associated with indicators of dental disease and psychosocial and structural factors, including gender identity, sexual orientation, suicidal ideation, challenges in accessing dental care and discrimination. Current evidence, though limited, suggests notable OHRQoL disparities among 2SLGBTQIAPN+ individuals. These findings highlight the need for more rigorous research and the development of inclusive oral health policies addressing the unique needs of 2SLGBTQIAPN+ populations.

## Introduction

Health disparities related to sexual orientation and gender identity have increasingly emerged as a critical area of inquiry in public health research. Sexual and gender minorities experience disproportionate health burdens, including heightened exposure to risk factors, adverse health outcomes and persistent barriers to accessing health care.^1^ These disparities are reflected in a higher prevalence of substance use disorders, mental health conditions, suicide attempts and suicide-related mortality, as well as elevated rates of tobacco use and binge or heavy alcohol consumption. In addition, these populations are less likely to utilise preventive health services and are disproportionately affected by certain infectious diseases.^1^ Collectively, these communities are often described using the umbrella term 2SLGBTQIAPN+, which encompasses individuals who identify as 2-spirit, lesbian, gay, bisexual, transgender, queer/questioning, intersex, asexual, pansexual, nonbinary and other diverse sexual and gender minorities.^2^^,^^3^

Access to health services, including oral health care, is an essential component of population health across all gender identities, racial groups, socioeconomic strata and sexual orientations. Despite this, sexual and gender minorities experience disproportionate exposure to structural stigma, discrimination and social marginalization, which can limit both access to and engagement with health care services, including dental care.^4^ As a result, routine preventive dental care may be delayed or avoided, contributing to unmet oral health needs and progression of oral diseases. Consistent with this, a previous review reported that 2SLGBTQ+ populations were more likely to seek dental care for emergency visits rather than for routine preventive visits.^5^

Emerging evidence reported that sexual and gender minorities face a substantial burden of oral disease, with particularly increased risk observed among transgender individuals. A recent scoping review indicated a higher prevalence of oral health conditions, such as periodontal disease and dental caries, among these populations and consistently higher decayed, missing and filled teeth scores among transgender individuals compared with cisgender counterparts.^5^ As oral health is essential to overall health and well-being, oral diseases significantly contribute to the global burden of chronic illness and can lead to significant pain, discomfort, difficulties in mastication, inadequate dietary intake and consequent nutritional deficiencies. These diseases can adversely impact an individual’s overall health and quality of life.^6^

Oral health-related quality of life (OHRQoL) is a complex, multidimensional concept that reflects how oral health impacts individuals’ comfort, daily functioning and well-being, including activities such as eating, sleeping and social interaction, as well as perceptions of self-worth and overall satisfaction with their oral health.^7^ As a patient-focused outcome, OHRQoL has gained growing prominence in dental public health for understanding the broader effects of oral disease and for evaluating the impact of oral health disparities on general health and quality of life.^8^

Although research on OHRQoL has significantly increased in recent decades, evidence concerning sexual and gender minorities remains limited and fragmented. The studies differ in their participant demographics, methodologies for assessing OHRQoL and reported outcomes, complicating comparisons among them. The scope and depth of research examining parameters related to OHRQoL vary considerably. These limitations demonstrate the importance of a thorough synthesis of existing evidence to clarify current understanding and identify gaps for future research. Therefore, the objective of this systematic review is to synthesise the current evidence on OHRQoL among sexual and gender minorities, as encompassed by the 2SLGBTQIAPN+ umbrella term.

## Methods

Before initiating the systematic review, a brief scoping search was performed to examine the current literature on the OHRQoL of 2SLGBTQIAPN+ and to extract relevant keywords from the literature. The review protocol was registered with the International Prospective Register of Systematic Reviews (CRD420251169537) as appropriate. The population, intervention, comparison, outcome method was initially used to formulate the review question. However, the intervention element was not defined because this systematic review intended to provide an overview of the OHRQoL for 2SLGBTQIAPN+, rather than to compare interventions for this population and the comparison was a non-2SLGBTQIAPN+ group if applicable. Consequently, the search strategy concentrated on identifying the population of interest as 2SLGBTQIAPN+ and the primary outcome, which is OHRQoL. This systematic review was reported in accordance with the preferred reporting items for systematic reviews and meta-analyses standards (Supplemental File 1).^9^

### Eligibility criteria

All primary study designs published in English were eligible for inclusion. Secondary literature (eg, reviews and related publications), guidelines and study protocols were excluded. Furthermore, case reports/series, books, book chapters, theses, dissertations, editorials, commentary papers, prefaces, conference proceedings, abstracts lacking full-text availability and purely qualitative studies were not eligible. Studies employing tools that assess general health-related quality of life (HRQoL) rather than oral health were not considered. Studies focusing on the development, cross-cultural adaptation or psychometric evaluation of OHRQoL tools (eg, validity and reliability testing) were also excluded. Additionally, studies involving participants with special health care needs, specific syndromes or diseases (eg, people living with HIV) or those related to specific medical or dental treatments were excluded to minimise confounding effects of disease-specific oral health conditions on OHRQoL outcomes. Studies focusing on specialised occupational, athletic, or high-vulnerability populations (eg, homeless individuals or self-harming adolescents) were excluded because their unique contextual and clinical characteristics may disproportionately impact OHRQoL, thereby limiting the applicability of findings to the target study population. Studies were eligible if they assessed OHRQoL in at least 1 subgroup within the 2SLGBTQIAPN+ populations using at least 1 validated OHRQoL assessment tool. Studies involving men who have sex with men (MSM) were also included, as MSM are commonly considered a sexual minority population. Only studies where the results for 2SLGBTQIAPN+ groups could be analysed independently were considered for inclusion if they had mixed populations.

### Information sources and search strategy

A comprehensive electronic literature search was initially conducted in November 2025, using 4 databases: EBSCOhost (dentistry and oral sciences source), PubMed, Scopus and Web of Science. The search was updated on 01 March 2026 during manuscript revision to identify any additional eligible studies. Google Scholar was also searched to capture grey literature; due to the large number of records retrieved, only the first 200 results ranked by relevance were screened. No restrictions were applied regarding publication year or geographic scope to ensure the inclusion of studies from diverse countries and time periods. The complete search strategies applied to each database are provided in Supplemental File 2. Additionally, the reference lists and citation tracking of the included studies were manually screened to identify additional relevant publications. All records were collected from electronic sources and managed using EndNote 21 bibliographic software.

### Selection process and data extraction

A 2-stage procedure was employed to select the studies, involving title/abstract screening followed by full-text screening. Three researchers (WMP, AM and PD) independently reviewed all studies during each of the 2 stages; if disagreements arose, they were discussed and/or resolved through consultation with additional researchers (DD or GH) as needed. In accordance with the preferred reporting items for systematic reviews and meta-analyses statement, the reasons for exclusion at the full-text stage were documented and reported (Figure).

**Fig:**
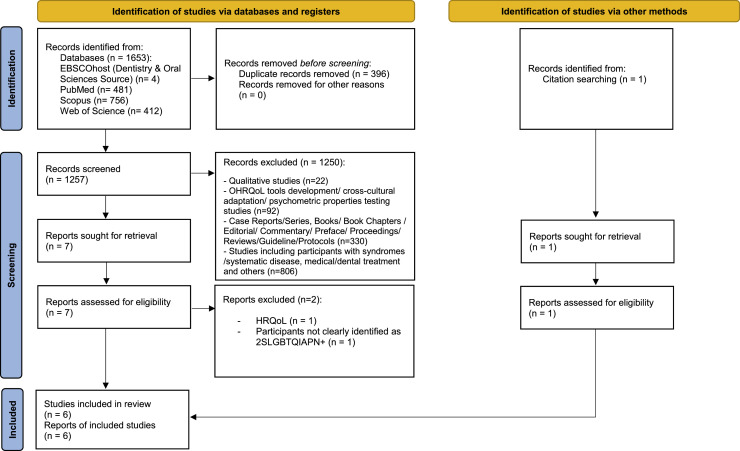
PRISMA 2020 flow diagram.

To gather pertinent data from all included research, a pretested data extraction spreadsheet was created. One researcher (WMP) extracted the data, which were then reviewed by 2 other researchers (AM and PD). Any differences were settled by consensus.

### Quality assessment

The methodological quality assessment was conducted using the Joanna Briggs Institute (JBI) Critical Appraisal Checklist for analytical cross-sectional studies^10^ by 1 researcher (WMP) and was then independently reviewed by 2 other researchers (AM and PD). This JBI appraisal checklist consists of 8 items evaluating: (Q1) clarity of inclusion criteria; (Q2) description of study subjects and setting; (Q3) validity and reliability of exposure measurement; (Q4) use of objective criteria for condition measurement; (Q5) identification of cofounding factors; (Q6) strategies to address cofounders; (Q7) validity and reliability of outcome measurement; and (Q8) appropriateness of statistical analysis. The possible respond options to each question were ‘yes’, ‘no’, ‘unclear’ and ‘not applicable’ as appropriate.

## Results

A total of 1653 potentially relevant studies were identified through the initial database searches. Following the elimination of duplicates, a total of 1257 studies were retained for the screening of titles and abstracts. The outcome of this process resulted in the identification of 7 studies for comprehensive evaluation of their full texts. After conducting a thorough evaluation of the full texts, 2 studies were excluded based on the established eligibility criteria: 1 study^11^ assessed general HRQoL rather than oral health among MSM in China and another study^12^ assessed OHRQoL among adolescents; however, the participants were not clearly identified as corresponding to at least 1 2SLGBTQIAPN+ subgroup. Furthermore, 1 study was identified through a manual review of the citation and reference lists of the included studies, resulting in a total of 6 studies included in this review. Figure presents a comprehensive flowchart that outlines the study selection process.

### Characteristics of the included studies

The general characteristics of the included studies are summarised in Table 1 and organised according to the OHRQoL tools employed. All studies adopted a cross-sectional analytical design, with 1 study conducted as an ancillary analysis of the Longitudinal Study of Australian Children. The studies were published between 2018 and 2025. Sample sizes ranged from 90 to 745 participants and the age of participants varied considerably across studies, spanning from adolescents aged 14 to 15 years to older adults up to 60+ years. Three studies were conducted in Brazil, while 1 study each was undertaken in India, Malaysia and Australia. Transgenders were the most frequently represented group in the included studies. One study focused exclusively on transgender women and another included transgenders and MSM. The remaining studies involved transgender participants together with other sexual and gender minorities such as lesbian, gay, bisexual, pansexual, asexual and queer individuals, except for 1 study that did not report the inclusion of transgender participants because information on gender identity was not available. All studies used validated OHRQoL tools administered either through self-administration or interviews in their respective languages, including Hindi, Malay, Portuguese and English. The Oral Health Impact Profile-14 (OHIP-14) was the most frequently used tool (3 of 6 studies), followed by the Oral Impacts on Daily Performances (OIDP) (2 of 6 studies), while the Pediatric Quality of Life Inventory (PedsQL) Oral Health Scale was used in 1 study.

**Table 1:** Characteristics of the included studies.

Study	Study design	Sample size and population	Age in y/range/mean (SD)	Sampling method	Study location and country	OHRQoL tool	OHRQoL response scale and score range	Negative OHRQoL impact classification	Language
Muralidharan et al.^13^	Cross-sectional	270 (MSMs: 241, TGs: 29)	18-54	Convenience sampling	Pune city, India	OHIP-14	5-point Likert (0-4)	NR	Hindi
Mohd et al.^14^	Cross-sectional	TGW: 100	Median (IQR): 32 (12)	Universal sampling	Terengganu state, Malaysia	OHIP-14	5-point Likert (0-4)	Code 2, 3 and 4	Malay
Almeida et al.^15^	Cross-sectional	464 (CGs: 328, TGs: 136)	18-70	Online voluntary nonprobability sampling	Online (Google Forms), Brazil	OHIP-14	5-point Likert (0-4)	Code 3 and 4	Portuguese
Prates et al.^16^	Controlled cross-sectional	90 (CGs: 45, TGs: 45)	18-44	Convenience sampling	Uberlândia, Minas Gerais, Brazil	OIDP (9 items) NOHS-B	Yes, no, don’t know/prefer not to answer	Yes	Portuguese
Guimarães et al.^17^	Cross-sectional	745 (CGs: 545, TGs/nonbinary/other: 200)	18-60+	Online voluntary nonprobability sampling	Online (Google Forms), Brazil	OIDP (9 items) NOHS-B	Yes, no, don’t know/prefer not to answer	Yes	Portuguese
Soares et al.^18^	Cross-sectional (ancillary study of the longitudinal study)	336 (LGBQ male: 106, LGBQ female: 230)	14-15 (LGBQ male: 178.3 (4.6) months, LGBQ female: 178.1 (4.6) months)	2-stage random sampling	Australia	PedsQ Oral Health Scale	5-point Likert (0-4)	Code 2, 3 and 4	English

CGs, cisgender; IQR, inter quartile range; LGBQ, lesbian, gay, bisexual, questioning; MSMs, men who have sex with men; NOHS-B, National Oral Health Survey of Brazil; NR, not reported; OHIP-14, Oral Health Impact Profile-14; OHRQoL, Oral Health Related Quality of Life; OIDP, Oral Impact on Daily Performance; PedsQ Oral Health Scale, Pediatric Quality of Life Inventory Oral Health Scale; SD, standard deviation; TGs, transgenders; TGW, transgender women.

### Oral health-related quality of life

Table 2 provides an overview of findings from studies employing different OHRQoL tools. The studies are organised according to the OHRQoL tools used and considerable variation was observed in the reporting of outcomes. While some studies reported overall mean scores with standard deviations or prevalence percentages, others presented both overall results and domain- or item-level findings, others reported only domain- or item-level findings, whereas some reported only domain- or item-level results. This variability highlights marked methodological heterogeneity in the assessment and reporting of OHRQoL within this population. In addition, only 2 studies reported OHRQoL outcomes separately according to sexual orientation among its study population.

**Table 2:** Negatively impacted oral health-related quality of life among participants in the included studies.

Study	OHRQoL tool	Overall mean (SD)/percentage	Mean (SD)/percentage of participants negatively impacted on their OHRQoL													
Muralidharan et al.^13^	OHIP-14	NR	Functional limitation		Physcial pain		Psychological discomfort		Physical disability		Psychological disability		Social disability		Handicap	
			Trouble pronouncing words	Taste worse	Painful aching	Uncomfortable to eat	Self-conscious	Tense	Diet unsatisfactory	Interrupt meals	Difficult to relax	Been embarrassed	Irritable with other	Difficulty doing jobs	Lifeless satisfying	Unable to function

35.6%	35.6%	35.6%	35.6%	36.7%	37.0%	36.3%	35.2%	36.3%	35.6%	35.6%	35.6%	36.3%	35.6%
Mohd et al.^14^	OHIP-14	16.67 (10.39)	Functional limitation		Physical pain		Psychological discomfort		Physical disability		Psychological disability		Social disability		Handicap	
			Difficulty chewing any food	Problem caused bad breath	Discomfort eating any food	Ulcers in mouth	Discomfort due to food getting stuck	Felt shy	Avoided eating certain foods	Avoided smiling	Sleep been disturbed	Concentration been disturbed	Avoided going out	Problems in carrying out daily activities	Had to spend a lot of money	Felt less confident
			1.14 (1.11)/34.6%	1.60 (1.33)/48.1%	1.45 (1.28)/46.1%	0.88 (0.96)/27.9%	2.50 (1.25)/76.9%	1.51 (1.49)/47.1%	1.45 (1.34)/47%	1.26 (1.49)/39.2%	0.88 (1.13)/29.8%	1.09 (1.11)/34.7%	0.49 (0.92)/13.9%	0.78 (1.04)/27.5%	0.73 (1.06)/20.6%	0.99 (1.29)/28.5%
Almeida et al.^15^	OHIP-14	33.2%	Functional limitation		Physical pain		Psychological discomfort		Physical disability		Psychological disability		Social disability		Handicap	
			6.9%		17.5%		27.8%		6.9%		18.3%		6.9%		6.7%	
Prates et al.^16^	OIDP		Impact on eating		Discomfort when brushing		Nervousness over teeth		Impact on leisure	Impact on sports practice	Speaking difficulty		Teeth cause shame	Teeth hinder studies	Impact on sleep	
		Transgender: NR	44.4%		37.8%		14.2%		20.0%	8.9%	15.6%		33.3%	13.3%	35.6%	
		LGBTQI+ cisgender: NR	35.6%		17.8%		11.1%		8.9%	2.2%	8.9%		6.7%	6.7%	15.6%	
Guimarães et al.^17^	OIDP	60.3%	Impact on eating		Teeth cleaning		Emotional state		Impact on leisure	Impact on sports practice	Speaking difficulty		Confidence to smile or speak	Interfere with studying or working	Impact on sleep	
			NR		33.9%		39.8%		NR	NR	NR		31.2%	NR	NR	
Soares et al.^18^	PedsQ Oral Health Scale		Tooth pain		Tooth pain when eating or drinking something cold, hot or sweet				Teeth that are dark in colour			Gum pain		Blood on the toothbrush after brushing		
		LGBQ male: 45.5%	NR		NR				NR			NR		NR		
		LGBQ female: 60.6 %	NR		NR				NR			NR		NR		

NR, not reported; OHIP-14, Oral Health Impact Profile-14; OHRQoL, Oral Health-Related Quality of Life; OIDP, Oral Impact on Daily Performance; PedsQ Oral Health Scale, Pediatric Quality of Life Inventory Oral Health Scale; SD, standard deviation.

### Oral Health Impact Profile-14

Three studies evaluated OHRQoL using the OHIP-14. Of these, 2 studies reported prevalence at the item level, whereas 1 study presented findings at the domain level. In a study conducted among MSM and transgender individuals in Pune, India, the psychological discomfort (feeling of tension) demonstrated the highest prevalence (37%).^13^ However, no statistically significant differences were observed across OHIP-14 domains and all domains were adversely affected by existing dental conditions. In a study of transgender women in Malaysia, the mean total OHIP-14 score was 16.67 (SD = 10.39).^14^ The least affected item was avoidance of going out, within the social disability domain (13.9%), whereas the most frequently reported item was psychological discomfort related to food impaction (76.9%). In this study, responses of ‘occasional’, ‘often’ or ‘very often’ were used to define the prevalence of oral health impacts. In an online survey of Brazilian adults identifying as LGBTIQ+, approximately one-third of participants (33.2%) reported a negative impact on their OHRQoL.^15^ The prevalence of OHRQoL impacts was 28.4% among cisgender participants and 44.8% among transgender participants. By sexual orientation, the prevalence of OHRQoL impacts was 40.5% among heterosexual participants, 29.2% among homosexual participants, 29.2% among bisexual participants, 37.5% among asexual participants, 48.5% among pansexual participants and 71.4% among other sexual and gender minority groups. The most frequently affected domains were psychological discomfort (27.8%), followed by psychological disability (18.3%) and physical pain (17.5%). In this study, the prevalence of oral health impacts was defined as the proportion of the participants who reported ‘fairly often’ or ‘very often’ responses to at least 1 OHIP-14 item.

### Oral impacts on daily performance

The following 2 Brazilian studies used the 9-item OIDP, consistent with the Brazilian National Oral Health Survey, with responses recorded as ‘Yes’, ‘No’ or ‘Don’t know/Prefer not to answer’. A study by Prates et al.^16^ involving LGBTQI+ populations, including transgender and cisgender groups, reported that oral health-related impacts on eating were the most prevalent in both groups (44.4% among transgender groups and 35.6% among cisgender groups), whereas impacts on sports participation were the least common (8.9% and 2.2%, respectively).^16^ Transgender groups had a higher prevalence of dental-related nervousness (Prevalence Ratio = 3.8; *P* = .002) and dental-related shame (Prevalence Ratio = 5.0; *P* = .033) compared with cisgender groups.

Another study conducted among LGBTQIA+ individuals through an online survey reported that the majority of participants (60.3%) experienced some impact of oral health on daily activities.^17^ Approximately 17% reported only 1 daily activity affected by oral health, while 15% reported impacts on 5 or more daily activities. Additionally, 44% of individuals with a positive self-perception of oral health reported at least 1 daily activity affected by oral health. Among those reporting impacts on OHRQoL, 67.1% were cisgender and 32.9% were transgender, nonbinary, or other gender identities; in terms of sexual orientation, 49.8% identified as homosexual, 41% as bisexual, asexual or other and 9.2% as heterosexual. The most frequently affected daily performance were emotional state, teeth cleaning and confidence to smile or speak.

### PedsQL Oral Health Scale

In the study among Australian adolescents, oral health-related impacts were reported by 43% of the study population.^18^ Overall, higher proportions of oral health impacts were observed among females compared with males and LGBQ adolescents demonstrated poorer oral health outcomes than their non-LGBQ counterparts. The prevalence of oral health impacts was 45.5% among LGBQ males and 60.6% among LGBQ females. In this study, responses of ‘sometimes’, ‘often’ and ‘almost always’ were categorised as indicating the presence of oral health impacts; however, item-level OHRQoL data were not reported.

### Factors related to the OHRQoL

Among the included studies, various factors associated with OHRQoL were identified. Indicators of dental disease, such as decayed and missing teeth and overall decayed, missing and filled teeth scores, were consistently linked to poor OHRQoL.^13^ Psychosocial factors, including gender identity, suicidal ideation, dissatisfaction with oral health status and challenges in accessing dental care, were also associated with impaired OHRQoL.^15^ Experiences of discrimination emerged as a significant determinant of oral health impacts, with the most significant associations consistently noted among LGBQ females.^18^ Another study also reported that sexual orientation (bisexual, asexual or other), higher levels of discrimination measured by the Everyday Discrimination Scale, experiences of discrimination in dental services, having the last dental appointment due to toothache, tooth extraction or treatment, self-perceived need for treatment and difficulty accessing dental services were associated with negative impacts on OHRQoL.^17^ In contrast, 1 study found no significant correlations between OHRQoL and sociodemographic factors,^14^ and this study, along with another^16^ did not examine oral health issues related to OHRQoL.

### Quality assessment

Five of the 8 quality criteria were met by all included studies. All studies clearly defined inclusion criteria and adequately described the study population and setting. Standardised criteria were consistently used for outcome measurement, indicating that outcomes were measured validly and reliably across all studies. In addition, all studies applied appropriate statistical analyses. However, exposure measurement was generally limited, with 5 studies rated as having inadequate validity and reliability and 1 study rated as unclear. Most studies identified potential confounding factors and applied appropriate strategies to control for confounding, while 2 studies did not address confounding. The detailed methodological quality of the included studies is presented in Table 3.

**Table 3:** Summary of quality assessment of included studies using the Joanna Briggs Institute Critical Appraisal Checklist for analytical cross-sectional studies.

Study	Q1	Q2	Q3	Q4	Q5	Q6	Q7	Q8
Muralidharan et al.^13^	Yes	Yes	Unclear	Yes	No	No	Yes	Yes
Mohd et al.^14^	Yes	Yes	No	Yes	Yes	Yes	Yes	Yes
Almeida et al.^15^	Yes	Yes	No	Yes	Yes	Yes	Yes	Yes
Prates et al.^16^	Yes	Yes	No	Yes	No	No	Yes	Yes
Guimarães et al.^17^	Yes	Yes	No	Yes	Yes	Yes	Yes	Yes
Soares et al.^18^	Yes	Yes	No	Yes	Yes	Yes	Yes	Yes

Q1: Were the criteria for inclusion in the sample clearly defined?

Q2: Were the study subjects and the setting described in detail?

Q3: Was the exposure measured in a valid and reliable way?

Q4: Were objective, standard criteria used for measurement of the condition?

Q5: Were confounding factors identified?

Q6: Were strategies to deal with confounding factors stated?

Q7: Were the outcomes measured in a valid and reliable way?

Q8: Was appropriate statistical analysis used?

## Discussion

This systematic review evaluated the existing evidence on OHRQoL within sexual and gender minorities, as represented by the 2SLGBTQIAPN+ umbrella term. Although there is an increasing awareness of oral health inequities among this population, only 6 studies met the eligibility criteria in this review, highlighting a substantial scarcity of OHRQOL-focused research in this area. This finding aligns with previous reviews reporting that research involving sexual and gender minorities in oral health is limited, fragmented and methodologically constrained.^5^^,^^19^ Taken together, the available evidence suggests that sexual and gender minorities experience measurable impacts on OHRQoL, although the magnitude and determinants of these impacts remain insufficiently understood due to the limited number and methodological diversity of existing studies. Nonetheless, while the included studies demonstrate that impacts on OHRQoL are present and multifaceted, they may be linked to the combined presence of clinical oral health conditions, psychosocial stressors and structural inequalities.

Although this review employs the inclusive umbrella term 2SLGBTQIAPN+ to facilitate the synthesis of literature, the included studies examined diverse populations, including MSM, transgender women, mixed LGBTIQ+ adult populations of cisgender and transgender individuals across various sexual orientations, LGBTQI+ cisgender and transgender groups and LGBQ adolescents. This diversity reflects both the evolution of terms used to describe sexual minority identities and the context in which the research was conducted. In contemporary literature regarding oral health, it should be emphasised that sexual orientation, gender identity and sex should not be treated as interchangeable or binary demographic variables, but as separate and intersecting social constructs linked to individuals’ health experiences and access to health care.^4^^,^^20^

Accordingly, the use of an umbrella term in this review aims to facilitate population-level synthesis rather than implying homogeneity of experiences or outcomes. Intersectionality frameworks highlight that oral health outcomes are shaped by the interplay of multiple social positions, such as gender identity, sexual orientation, race and socioeconomic status, within broader structural systems of inequity.^21^ In oral health research, this perspective is particularly relevant for understanding how intersecting social identities relate to differences in exposure to discrimination, access to health care resources and vulnerability to oral health problems.^22^ However, the inconsistent subgroup reporting and cross-sectional designs of included studies limited the ability to explore these intersections comprehensively. As such, the findings should be interpreted as indicative of general patterns rather than definitive subgroup-specific conclusions.

Patterns of oral health-related impacts varied across studies and appear to relate to differences in the OHRQoL tools used. In studies utilising the OHIP-14, psychological discomfort frequently emerged as one of the most impacted domains, particularly when domain- or item-level findings were reported; however, not all studies identified statistically significant differences between domains.^13^, ^14^, ^15^ Studies using the OIDP highlighted functional and emotional impacts as the most commonly reported outcomes.^16^^,^^17^ Only 1 study was constrained for domain-level interpretation owing to insufficient detailed reporting.^18^ These findings suggest that both psychological and functional aspects are salient components of OHRQoL among sexual and gender minorities, while also underscoring the impact of measurement methods on recognising these differences.

Clinical oral disease burden appears to be an important factor related to impaired OHRQoL, consistent with longstanding evidence linking untreated oral conditions with diminished quality of life.^6^^,^^8^ This perspective highlights how untreated dental disease relates to differences in daily functioning, discomfort and social participation, which are central components captured by OHRQoL measures. However, the limited integration of clinical assessments with OHRQoL measures highlights an important evidence gap in research involving sexual and gender minorities.^19^ Without concurrent clinical data, it remains difficult to fully understand how objective oral health conditions correspond with perceived quality of life impacts within these populations.

In addition to clinical disease burden, OHRQoL is associated with broader psychosocial and structural contexts, including access to care and experiences within health care settings. Structural discrimination may be manifested in institutional barriers, policy frameworks and organisational practices linked to unequal access to health care among sexual and gender minorities.^23^ These patterns are consistent with existing evidence suggesting that discrimination, financial barriers and lack of culturally safe dental care are related to unmet oral health needs and irregular service use among this population.^5^^,^^24^ Such structural conditions may also relate to both disease burden and psychosocial stressors, thereby reinforcing inequities in oral health outcomes and perceived OHRQoL.

The relationship between discrimination and poorer OHRQoL may be interpreted in light of Minority Stress Theory, which suggests that stigma and social exclusion experienced by sexual and gender minorities function as chronic stressors linked to adverse health outcomes.^25^ Within this framework, psychosocial stressors such as stigma, expectations of rejection and identity concealment may be related to both health behaviours and subjective perceptions of well-being.^26^ Discrimination in an oral healthcare setting may be associated with reduced dental service utilisation, delayed treatment and increased dental anxiety; together, these factors may be linked to poorer oral health and perception of oral health.^5^^,^^24^ As OHRQoL tools capture subjective experiences such as embarrassment, discomfort and social interference, they are particularly sensitive to the psychological dimensions of minority stress and may help to contextualise the prominence of psychosocial impacts observed in some of the included studies.

Substantial heterogeneity in OHRQoL measurement and reporting was observed across the included studies, including differences in tools used, outcome metrics and reporting styles. Among studies using the OHIP-14, variation was noted in how negative OHRQoL impacts were operationalised (ie, using 2 vs 3 response categories), which is associated with differences in prevalence estimates and may limit direct comparability across studies. Differences in item-to-domain allocation were also identified among OHIP-14 studies,^13^^,^^14^ indicating that domain-level comparisons, especially across culturally distinct settings, should be interpreted with caution. Although cross-cultural translation and adaptation of OHRQoL tools aim to ensure linguistic and cultural relevance, such adaptations may also reflect differences in how oral health impacts are perceived across cultures and populations.^27^

Several limitations should be considered when interpreting the findings of this review. First, the search strategy was restricted to English-language publications, which may have resulted in the exclusion of relevant studies published in other languages, although the extent of non-English literature on this topic remains unclear. Second, grey literature was searched using Google Scholar and manual reference screening; however, some relevant nonindexed studies may not have been identified. Additionally, the screening of Google Scholar results was limited to the first 200 most relevant records; therefore, some potentially relevant studies may have been missed. Third, given the higher prevalence of certain health conditions, including HIV, among sexual and gender minorities, the exclusion of disease-specific studies may have limited the inclusion of potentially relevant evidence. Furthermore, all included studies were cross-sectional, limiting causal inference and generalisability; therefore, the findings should be interpreted as associations rather than causal relationships. A meta-analysis was not conducted due to substantial heterogeneity in study populations, OHRQoL tools and scoring systems, highlighting the need for greater standardisation and transparency in OHRQoL measurement and reporting. In addition, outcomes were reported using different formats, limiting comparability across studies. Pooling these results could therefore produce misleading summary estimates. Despite these limitations, this review provides a synthesis of the available evidence on OHRQoL among sexual and gender minorities and highlights important gaps for future research.

Despite increasing legal recognition of sexual and gender diversity in many countries, including the approval of same-sex marriage and the adoption of inclusive policies, the limited number of eligible studies identified in this review indicates that OHRQoL remains an underexplored outcome. This gap suggests that broader social progress has not yet resulted in a sufficiently robust evidence base to guide oral health equity for sexual and gender minorities. Another issue is that existing OHRQoL tools were developed for general populations and may not fully capture dimensions particularly relevant to sexual and gender minorities, such as stigma-related distress or negative health care experiences. Accordingly, there have been calls for oral health research to move beyond binary and decontextualised measures towards more inclusive, person-centered approaches, including the development and validation of OHRQoL tools tailored to these populations.^28^

Overall, these findings highlight the need for equity-focused dental public health strategies that address both clinical needs and the broader social determinants of oral health. Disparities in OHRQoL among sexual and gender minorities appear to be associated with multiple factors, including clinical disease burden, psychosocial stressors, structural discrimination and challenges in accessing oral health care. Expanding research on OHRQoL is essential to better understand its impact on well-being in these populations. Strengthening education and training for the oral health workforce, including content on sexual orientation, gender identity and culturally safe and inclusive care, may improve provider competence, reduce stigma in clinical settings and enhance patient-provider interactions. Future studies should adopt longitudinal and intersectional methodologies, integrate clinical and patient-reported outcomes and differentiate findings by sexual orientation and gender identity to clarify the relationship between oral health and OHRQoL. Such efforts are essential for advancing oral health equity and informing evidence-based policy and practice.

## Conclusion

In conclusion, this systematic review highlights that OHRQoL among sexual and gender minorities is associated with oral health conditions, psychosocial stressors and structural inequities such as discrimination and challenges in accessing oral health services. Future research should adopt more robust and inclusive approaches to better understand OHRQoL in this population and to inform equitable oral health policies and dental public health practices that recognise the complex social contexts in which oral health is experienced.

## Conflict of interest

None disclosed.

## Appendix Supplementary materials

Image, application 1

Image, application 2

## Footnotes

Supplementary material associated with this article can be found in the online version at doi:10.1016/j.identj.2026.109624.
