# Supplementary material for: Oral Health-related Quality of Life Among 2SLGBTQIAPN+: A Systematic Review
Source: Int Dent J. 2026 May 15;76(4):109624. doi: 10.1016/j.identj.2026.109624 (PMC13202278; doi:10.1016/j.identj.2026.109624)
Supplement: Supplementary file 2 [file mmc2.docx]

**Search Strategies used for each database**

**EBSCOhost (Dentistry & Oral Sciences Source)**

| (“sexual minority” OR “gender minority” OR “sexual and gender minorities” OR “gender-expansive” OR “gender diverse” OR genderqueer OR demisexual OR eunuch OR “third gender” OR “fluid gender” OR LGBT OR LGBTQ+ OR 2SLGBTQ+ OR LGBTQIA+ OR LGBTQIAPN+ OR two-spirit OR lesbian OR gay OR bisexual OR transgender OR transmen OR transwomen OR queer OR questioning OR intersex OR asexual OR pansexual OR non-binary OR homosexual OR “men who have sex with men” OR MSM OR “women who have sex with women” OR WSW) (All Fields) |
| --- |
| AND |
| (“oral health-related quality of life” OR OHRQoL) (All fields) |

**PubMed**

| (“sexual minority” OR “gender minority” OR “sexual and gender minorities” OR “gender-expansive” OR “gender diverse” OR genderqueer OR demisexual OR eunuch OR “third gender” OR “fluid gender” OR LGBT OR LGBTQ+ OR 2SLGBTQ+ OR LGBTQIA+ OR LGBTQIAPN+ OR two-spirit OR lesbian OR gay OR bisexual OR transgender OR transmen OR transwomen OR queer OR questioning OR intersex OR asexual OR pansexual OR non-binary OR homosexual OR “men who have sex with men” OR MSM OR “women who have sex with women” OR WSW) |
| --- |
| AND |
| (“oral health-related quality of life” OR OHRQoL) |

**Scopus**

| ( ALL ( "sexual minority" ) OR ALL ( "gender minority" ) OR (“sexual and gender minorities”) OR ALL ( "gender-expansive" ) OR ALL ( "gender diverse" ) OR ALL ( genderqueer ) OR ALL ( demisexual ) OR ALL ( eunuch ) OR ALL ( "third gender" ) OR ALL ( "fluid gender" ) OR ALL ( LGBT ) OR ALL ( LGBTQ+ ) OR ALL ( 2SLGBTQ+ ) OR ALL ( LGBTQIA+ ) OR ALL ( LGBTQIAPN+ ) OR ALL ( two-spirit ) OR ALL ( lesbian ) OR ALL ( gay ) OR ALL ( bisexual ) OR ALL ( transgender ) OR ALL ( transmen ) OR ALL ( transwomen ) OR ALL ( queer ) OR ALL ( questioning ) OR ALL ( intersex ) OR ALL ( asexual ) OR ALL ( pansexual ) OR ALL ( non-binary ) OR ALL ( homosexual ) OR ALL ( "men who have sex with men" ) OR ALL ( msm ) OR ALL ( "women who have sex with women" ) OR ALL ( WSW ) |
| --- |
| AND |
| ALL ( "oral health-related quality of life" ) OR ALL ( OHRQoL ) ) |

**Web of Science**

| (“sexual minority” OR “gender minority” OR “sexual and gender minorities” OR “gender-expansive” OR “gender diverse” OR genderqueer OR demisexual OR eunuch OR “third gender” OR “fluid gender” OR LGBT OR LGBTQ+ OR 2SLGBTQ+ OR LGBTQIA+ OR LGBTQIAPN+ OR two-spirit OR lesbian OR gay OR bisexual OR transgender OR transmen OR transwomen OR queer OR questioning OR intersex OR asexual OR pansexual OR non-binary OR homosexual OR “men who have sex with men” OR MSM OR “women who have sex with women” OR WSW) (All Fields) |
| --- |
| AND |
| (“oral health-related quality of life” OR OHRQoL) (All fields) |

**Google Scholar (only the first 200 results ranked by relevance were screened)**

| (“sexual minority” OR “gender minority” OR “sexual and gender minorities” OR “gender-expansive” OR “gender diverse” OR genderqueer OR demisexual OR eunuch OR “third gender” OR “fluid gender” OR LGBT OR LGBTQ+ OR 2SLGBTQ+ OR LGBTQIA+ OR LGBTQIAPN+ OR two-spirit OR lesbian OR gay OR bisexual OR transgender OR transmen OR transwomen OR queer OR questioning OR intersex OR asexual OR pansexual OR non-binary OR homosexual OR “men who have sex with men” OR MSM OR “women who have sex with women” OR WSW) AND (“oral health-related quality of life”) |
| --- |
